# *N*,*N*′-Diallyl-2,2′,5,5′-tetra­chloro-*N*,*N*′-[1,3-phenyl­enebis(methyl­ene)]dibenzene­sulfonamide

**DOI:** 10.1107/S1600536811045478

**Published:** 2011-11-12

**Authors:** Tahir Ali Sheikh, Ejaz, Islam Ullah Khan, William T. A. Harrison

**Affiliations:** a Materials Chemistry Laboratry, Department of Chemistry, GC University, Lahore 54000, Pakistan; b Department of Chemistry, University of Aberdeen, Meston Walk, Aberdeen AB24 3UE, Scotland

## Abstract

In the title compound, C_26_H_24_Cl_4_N_2_O_4_S_2_, the dihedral angles between the central benzene ring and the pendant rings are 70.07 (12) and 59.07 (12)°. The equivalent angle between the pendant rings is 79.24 (12)°. Both sulfonamide groups lie to the same side of the central ring but the pendant chains have very different conformations, as indicated by their C—S—N—C torsion angles [104.66 (17) and −76.35 (19)°] and S—N—C—C torsion angles [129.61 (17) and 147.10 (17)°]. Both N atoms are close to planar (bond angle sums = 359.0 and 354.8°). In the crystal, inversion dimers are formed *via* a pair of weak C—H⋯O inter­actions which generate *R*_2_^2^(22) loops.

## Related literature

For related structures, see: Ejaz *et al.* (2011*a* ▶,*b* ▶).
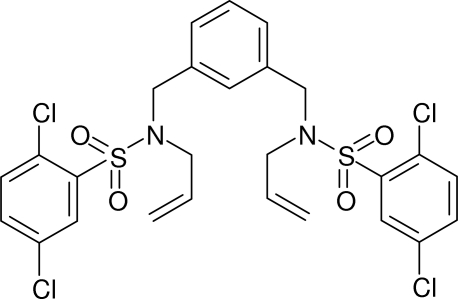


## Experimental

### 

#### Crystal data

C_26_H_24_Cl_4_N_2_O_4_S_2_
*M* *_r_* = 634.39
Triclinic, 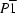

*a* = 8.2744 (2) Å
*b* = 11.3398 (2) Å
*c* = 15.6481 (4) Å
α = 87.777 (1)°
β = 84.443 (1)°
γ = 84.257 (1)°
*V* = 1453.40 (6) Å^3^
*Z* = 2
Mo *K*α radiation
μ = 0.59 mm^−1^
*T* = 296 K
0.40 × 0.15 × 0.15 mm

#### Data collection

Bruker APEXII CCD diffractometer
21435 measured reflections
7190 independent reflections
5200 reflections with *I* > 2σ(*I*)
*R*_int_ = 0.025

#### Refinement

*R*[*F* ^2^ > 2σ(*F* ^2^)] = 0.044
*wR*(*F* ^2^) = 0.122
*S* = 1.05
7190 reflections
343 parameters
H-atom parameters constrained
Δρ_max_ = 0.65 e Å^−3^
Δρ_min_ = −0.59 e Å^−3^

### 

Data collection: *APEX2* (Bruker, 2007 ▶); cell refinement: *SAINT* (Bruker, 2007 ▶); data reduction: *SAINT*; program(s) used to solve structure: *SHELXS97* (Sheldrick, 2008 ▶); program(s) used to refine structure: *SHELXL97* (Sheldrick, 2008 ▶); molecular graphics: *ORTEP-3* (Farrugia, 1997 ▶); software used to prepare material for publication: *SHELXL97*.

## Supplementary Material

Crystal structure: contains datablock(s) I, global. DOI: 10.1107/S1600536811045478/su2341sup1.cif

Structure factors: contains datablock(s) I. DOI: 10.1107/S1600536811045478/su2341Isup2.hkl

Supplementary material file. DOI: 10.1107/S1600536811045478/su2341Isup3.cml

Additional supplementary materials:  crystallographic information; 3D view; checkCIF report

## Figures and Tables

**Table 1:** Hydrogen-bond geometry (Å, °)

*D*—H⋯*A*	*D*—H	H⋯*A*	*D*⋯*A*	*D*—H⋯*A*
C8—H8*A*⋯O3^i^	0.97	2.59	3.422 (3)	144

Symmetry code: (i) 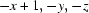.

**Fig. 1.**
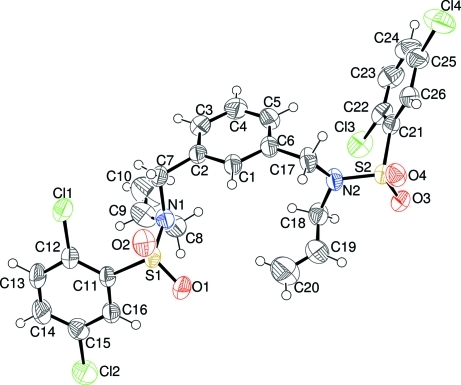
The molecular structure of the title molecule, showing 50% displacement ellipsoids and the atom numbeirng scheme

**Fig. 2.**
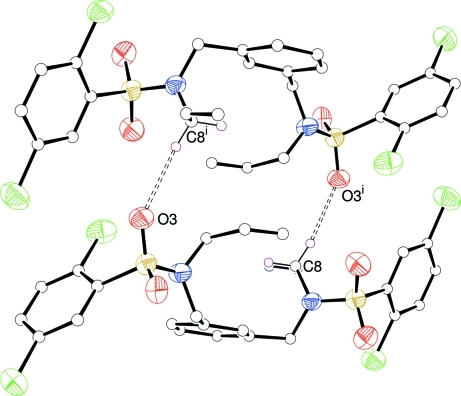
A view of the C—H···O hydrogen-bond (double-dashed line) inversion dimer in the crystal of the title compound [C atoms are shown as spheres; H atoms, except those attached to C8, have been omitted for clarity; symmetry code: (i) –x+1, –y, –z].

## supplementary crystallographic information

### Comment

As part of our ongoing structural studies of symmetrical aryl sulfonamides (Ejaz *et al.*, 2011*a*,*b*), the synthesis and structure of the title compound, (I), (Fig. 1), are now described.

The dihedral angles between the central (C1-C6) benzene ring and the pendant (C11-C16) and (C2-C26)1 rings are 70.07 (12) and 59.07 (12)°, respectively. The equivalent angle between the pendant rings is 79.24 (12)°. Both sulfonamide groups lie to the same side of the central ring but the pendant chains have very different conformations, as indicated by their C11—S1—N1—C7 and C21—S2—N2—C17 torsion angles [104.66 (17) and -76.35 (19)°, respectively] and their S1—N1—C7—C2 and S2—N2—C17—C6 torsion angles [129.61 (17) and 147.10 (17)°, respectively]. The N atoms are close to planar (bond angle sums = 359.0 and 354.8°).

In the crystal, inversion dimers are formed, by a pair of weak C—H···O interactions which generate *R*^2^_2_(22) loops (Fig. 2). There are no significant aromatic π-π stacking interactions in the crystal.

### Experimental

A mixture of *N*,*N*'-(benzene-1,3-diyldimethanediyl)bis(2,5-dichlorobenzenesulfonamide) (0.32 g; 0.5 mmol), sodium hydride (0.25 g; 0.9 mmol) and *N*,*N*-dimethylformamide (10.0 ml) was stirred in a 100-ml round bottom flask at room temperature for half an hour, followed by the addition of allyl bromide (0.1 ml, 1.0 mmol). The reaction mixture was further stirred for five hours, and its completion was monitored by TLC. After completion, the contents were poured over crushed ice. The precipitated product was isolated, washed and recrystallized from methanol to yield colourless block-like crystals of the title compound.

### Refinement

The hydrogen atoms were placed in calculated positions (C—H = 0.93–0.97 Å) and refined as riding atoms with *U*_iso_(H) = 1.2*U*_eq_(C) or 1.5*U*_eq_(methyl C). The methyl groups were allowed to rotate, but not to tip, to best fit the electron density.

### Crystal data

**Table:**

C_26_H_24_Cl_4_N_2_O_4_S_2_	*Z* = 2
*M**_r_* = 634.39	*F*(000) = 652
Triclinic, *P*1	*D*_x_ = 1.450 Mg m^−^^3^
Hall symbol: -P 1	Mo *K*α radiation, λ = 0.71073 Å
*a* = 8.2744 (2) Å	Cell parameters from 7190 reflections
*b* = 11.3398 (2) Å	θ = 2.2–26.4°
*c* = 15.6481 (4) Å	µ = 0.59 mm^−^^1^
α = 87.777 (1)°	*T* = 296 K
β = 84.443 (1)°	Block, colourless
γ = 84.257 (1)°	0.40 × 0.15 × 0.15 mm
*V* = 1453.40 (6) Å^3^	

### Data collection

**Table:**

Bruker APEXII CCD diffractometer	5200 reflections with *I* > 2σ(*I*)
Radiation source: fine-focus sealed tube	*R*_int_ = 0.025
graphite	θ_max_ = 28.4°, θ_min_ = 2.9°
ω scans	*h* = −11→11
21435 measured reflections	*k* = −15→15
7190 independent reflections	*l* = −20→20

### Refinement

**Table:**

Refinement on *F*^2^	Primary atom site location: structure-invariant direct methods
Least-squares matrix: full	Secondary atom site location: difference Fourier map
*R*[*F*^2^ > 2σ(*F*^2^)] = 0.044	Hydrogen site location: inferred from neighbouring sites
*wR*(*F*^2^) = 0.122	H-atom parameters constrained
*S* = 1.05	*w* = 1/[σ^2^(*F*_o_^2^) + (0.0515*P*)^2^ + 0.5046*P*] where *P* = (*F*_o_^2^ + 2*F*_c_^2^)/3
7190 reflections	(Δ/σ)_max_ < 0.001
343 parameters	Δρ_max_ = 0.65 e Å^−^^3^
0 restraints	Δρ_min_ = −0.59 e Å^−^^3^

### Special details

**Table:**

Geometry. All e.s.d.'s (except the e.s.d. in the dihedral angle between two l.s. planes) are estimated using the full covariance matrix. The cell e.s.d.'s are taken into account individually in the estimation of e.s.d.'s in distances, angles and torsion angles; correlations between e.s.d.'s in cell parameters are only used when they are defined by crystal symmetry. An approximate (isotropic) treatment of cell e.s.d.'s is used for estimating e.s.d.'s involving l.s. planes.
Refinement. Refinement of *F*^2^ against ALL reflections. The weighted *R*-factor *wR* and goodness of fit *S* are based on *F*^2^, conventional *R*-factors *R* are based on *F*, with *F* set to zero for negative *F*^2^. The threshold expression of *F*^2^ > σ(*F*^2^) is used only for calculating *R*-factors(gt) *etc*. and is not relevant to the choice of reflections for refinement. *R*-factors based on *F*^2^ are statistically about twice as large as those based on *F*, and *R*- factors based on ALL data will be even larger.

### Fractional atomic coordinates and isotropic or equivalent isotropic displacement parameters (Å^2^)

**Table:**

	*x*	*y*	*z*	*U*_iso_*/*U*_eq_	
C1	0.3523 (3)	0.37348 (17)	0.05525 (13)	0.0427 (5)	
H1	0.4634	0.3801	0.0553	0.051*	
C2	0.2551 (3)	0.36986 (17)	0.13297 (13)	0.0436 (5)	
C3	0.0903 (3)	0.3619 (2)	0.13202 (16)	0.0567 (6)	
H3	0.0238	0.3608	0.1834	0.068*	
C4	0.0236 (3)	0.3556 (3)	0.05513 (19)	0.0681 (7)	
H4	−0.0878	0.3500	0.0549	0.082*	
C5	0.1202 (3)	0.3573 (2)	−0.02092 (17)	0.0630 (7)	
H5	0.0742	0.3518	−0.0724	0.076*	
C6	0.2857 (3)	0.36727 (18)	−0.02203 (13)	0.0475 (5)	
C7	0.3328 (3)	0.36645 (18)	0.21600 (13)	0.0452 (5)	
H7A	0.3917	0.4358	0.2188	0.054*	
H7B	0.2492	0.3673	0.2640	0.054*	
C8	0.3762 (3)	0.14402 (19)	0.22185 (16)	0.0560 (6)	
H8A	0.4636	0.0801	0.2193	0.067*	
H8B	0.3180	0.1408	0.1712	0.067*	
C9	0.2634 (4)	0.1266 (2)	0.2993 (2)	0.0722 (8)	
H9	0.3045	0.1303	0.3522	0.087*	
C10	0.1167 (5)	0.1073 (4)	0.2996 (3)	0.1118 (13)	
H10A	0.0708	0.1030	0.2480	0.134*	
H10B	0.0538	0.0974	0.3515	0.134*	
C11	0.6357 (2)	0.20232 (19)	0.35234 (12)	0.0414 (4)	
C12	0.5655 (3)	0.2651 (2)	0.42343 (13)	0.0469 (5)	
C13	0.5809 (3)	0.2177 (2)	0.50477 (14)	0.0616 (6)	
H13	0.5360	0.2610	0.5520	0.074*	
C14	0.6615 (4)	0.1076 (3)	0.51736 (16)	0.0670 (7)	
H14	0.6721	0.0766	0.5726	0.080*	
C15	0.7263 (3)	0.0438 (2)	0.44686 (15)	0.0550 (6)	
C16	0.7139 (3)	0.0899 (2)	0.36483 (14)	0.0474 (5)	
H16	0.7579	0.0457	0.3178	0.057*	
C17	0.3948 (3)	0.36633 (19)	−0.10491 (13)	0.0529 (6)	
H17A	0.3550	0.4297	−0.1432	0.064*	
H17B	0.5044	0.3799	−0.0934	0.064*	
C18	0.4535 (3)	0.14377 (19)	−0.09764 (15)	0.0547 (6)	
H18A	0.4232	0.0750	−0.1250	0.066*	
H18B	0.3977	0.1464	−0.0402	0.066*	
C19	0.6323 (4)	0.1299 (3)	−0.09123 (19)	0.0726 (8)	
H19	0.6998	0.1201	−0.1420	0.087*	
C20	0.7008 (5)	0.1304 (3)	−0.0213 (3)	0.1042 (12)	
H20A	0.6375	0.1400	0.0308	0.125*	
H20B	0.8140	0.1212	−0.0226	0.125*	
C21	0.2219 (3)	0.30691 (18)	−0.27923 (13)	0.0428 (5)	
C22	0.0825 (3)	0.2487 (2)	−0.25724 (16)	0.0539 (6)	
C23	−0.0632 (3)	0.2931 (3)	−0.2852 (2)	0.0703 (8)	
H23	−0.1553	0.2530	−0.2710	0.084*	
C24	−0.0753 (3)	0.3962 (3)	−0.3342 (2)	0.0723 (8)	
H24	−0.1748	0.4257	−0.3533	0.087*	
C25	0.0607 (3)	0.4551 (2)	−0.35453 (17)	0.0591 (6)	
C26	0.2105 (3)	0.41098 (19)	−0.32823 (14)	0.0468 (5)	
H26	0.3024	0.4509	−0.3433	0.056*	
S1	0.63115 (7)	0.25666 (5)	0.24397 (3)	0.04469 (14)	
S2	0.41781 (6)	0.25211 (5)	−0.25007 (3)	0.04245 (14)	
N1	0.4463 (2)	0.25790 (15)	0.22148 (11)	0.0450 (4)	
N2	0.3983 (2)	0.25128 (15)	−0.14661 (11)	0.0485 (4)	
O1	0.7286 (2)	0.16884 (15)	0.19430 (10)	0.0610 (4)	
O2	0.6734 (2)	0.37546 (14)	0.23770 (10)	0.0586 (4)	
O3	0.4524 (2)	0.13258 (14)	−0.27629 (10)	0.0560 (4)	
Cl1	0.45440 (9)	0.40108 (6)	0.41341 (4)	0.06439 (18)	
Cl2	0.82694 (11)	−0.09625 (7)	0.45968 (5)	0.0831 (2)	
Cl3	0.08862 (9)	0.12043 (6)	−0.19397 (5)	0.0722 (2)	
Cl4	0.04722 (11)	0.58805 (7)	−0.41244 (6)	0.0902 (3)	
O4	0.52548 (18)	0.33697 (15)	−0.28380 (10)	0.0525 (4)	

### Atomic displacement parameters (Å^2^)

**Table:**

	*U*^11^	*U*^22^	*U*^33^	*U*^12^	*U*^13^	*U*^23^
C1	0.0560 (13)	0.0353 (10)	0.0367 (10)	−0.0047 (9)	−0.0038 (9)	−0.0011 (8)
C2	0.0565 (13)	0.0350 (10)	0.0379 (11)	0.0030 (9)	−0.0044 (9)	−0.0015 (8)
C3	0.0557 (14)	0.0599 (14)	0.0517 (14)	0.0045 (11)	0.0003 (11)	−0.0044 (11)
C4	0.0500 (14)	0.0834 (19)	0.0707 (18)	0.0048 (13)	−0.0112 (13)	−0.0129 (14)
C5	0.0723 (17)	0.0652 (16)	0.0524 (14)	0.0078 (13)	−0.0229 (13)	−0.0080 (12)
C6	0.0699 (15)	0.0351 (10)	0.0371 (11)	−0.0007 (10)	−0.0072 (10)	−0.0013 (8)
C7	0.0587 (13)	0.0416 (11)	0.0335 (10)	−0.0001 (9)	0.0011 (9)	−0.0039 (8)
C8	0.0685 (15)	0.0386 (11)	0.0630 (15)	−0.0089 (10)	−0.0144 (12)	0.0006 (10)
C9	0.0738 (19)	0.0669 (17)	0.080 (2)	−0.0249 (14)	−0.0178 (15)	0.0206 (14)
C10	0.083 (2)	0.132 (3)	0.127 (3)	−0.044 (2)	−0.020 (2)	0.029 (3)
C11	0.0436 (11)	0.0504 (12)	0.0306 (10)	−0.0091 (9)	−0.0017 (8)	−0.0018 (8)
C12	0.0524 (12)	0.0527 (12)	0.0363 (11)	−0.0080 (10)	−0.0027 (9)	−0.0050 (9)
C13	0.0766 (17)	0.0754 (17)	0.0310 (11)	−0.0037 (14)	0.0019 (11)	−0.0058 (11)
C14	0.0852 (19)	0.0777 (18)	0.0365 (12)	−0.0043 (15)	−0.0051 (12)	0.0085 (12)
C15	0.0603 (14)	0.0558 (14)	0.0485 (13)	−0.0063 (11)	−0.0069 (11)	0.0089 (10)
C16	0.0489 (12)	0.0559 (13)	0.0379 (11)	−0.0069 (10)	−0.0035 (9)	−0.0033 (9)
C17	0.0875 (18)	0.0387 (11)	0.0334 (11)	−0.0118 (11)	−0.0048 (11)	0.0005 (8)
C18	0.0774 (17)	0.0414 (12)	0.0432 (12)	−0.0038 (11)	0.0008 (11)	0.0045 (9)
C19	0.081 (2)	0.0750 (18)	0.0587 (16)	0.0039 (15)	−0.0086 (14)	0.0118 (14)
C20	0.105 (3)	0.114 (3)	0.096 (3)	0.001 (2)	−0.032 (2)	−0.004 (2)
C21	0.0441 (11)	0.0470 (11)	0.0377 (11)	−0.0051 (9)	−0.0011 (9)	−0.0109 (9)
C22	0.0494 (13)	0.0528 (13)	0.0593 (14)	−0.0087 (10)	0.0041 (11)	−0.0141 (11)
C23	0.0453 (14)	0.0733 (18)	0.093 (2)	−0.0101 (12)	−0.0023 (13)	−0.0180 (16)
C24	0.0486 (14)	0.0769 (19)	0.093 (2)	0.0034 (13)	−0.0200 (14)	−0.0187 (16)
C25	0.0648 (16)	0.0526 (14)	0.0609 (15)	0.0052 (12)	−0.0172 (12)	−0.0116 (11)
C26	0.0500 (12)	0.0480 (12)	0.0436 (12)	−0.0043 (9)	−0.0085 (9)	−0.0083 (9)
S1	0.0529 (3)	0.0501 (3)	0.0300 (2)	−0.0060 (2)	0.0017 (2)	−0.0006 (2)
S2	0.0450 (3)	0.0478 (3)	0.0337 (3)	−0.0032 (2)	0.0003 (2)	−0.0033 (2)
N1	0.0604 (11)	0.0355 (9)	0.0401 (9)	−0.0053 (8)	−0.0098 (8)	−0.0001 (7)
N2	0.0749 (13)	0.0377 (9)	0.0324 (9)	−0.0040 (8)	−0.0041 (8)	−0.0002 (7)
O1	0.0690 (11)	0.0715 (11)	0.0369 (8)	0.0091 (9)	0.0071 (7)	−0.0046 (7)
O2	0.0689 (11)	0.0584 (10)	0.0504 (9)	−0.0231 (8)	−0.0005 (8)	0.0057 (7)
O3	0.0645 (10)	0.0540 (9)	0.0468 (9)	0.0077 (8)	−0.0003 (7)	−0.0138 (7)
Cl1	0.0808 (4)	0.0610 (4)	0.0484 (3)	0.0087 (3)	−0.0027 (3)	−0.0135 (3)
Cl2	0.1044 (6)	0.0696 (4)	0.0707 (5)	0.0120 (4)	−0.0131 (4)	0.0149 (4)
Cl3	0.0726 (4)	0.0601 (4)	0.0836 (5)	−0.0233 (3)	0.0114 (4)	−0.0002 (3)
Cl4	0.1033 (6)	0.0672 (4)	0.1033 (6)	0.0049 (4)	−0.0444 (5)	0.0105 (4)
O4	0.0452 (8)	0.0693 (10)	0.0431 (8)	−0.0114 (7)	−0.0023 (7)	0.0070 (7)

### Geometric parameters (Å, °)

**Table:**

C1—C6	1.384 (3)	C15—C16	1.376 (3)
C1—C2	1.393 (3)	C15—Cl2	1.732 (3)
C1—H1	0.9300	C16—H16	0.9300
C2—C3	1.378 (3)	C17—N2	1.478 (3)
C2—C7	1.502 (3)	C17—H17A	0.9700
C3—C4	1.379 (4)	C17—H17B	0.9700
C3—H3	0.9300	C18—N2	1.472 (3)
C4—C5	1.369 (4)	C18—C19	1.484 (4)
C4—H4	0.9300	C18—H18A	0.9700
C5—C6	1.383 (4)	C18—H18B	0.9700
C5—H5	0.9300	C19—C20	1.281 (4)
C6—C17	1.507 (3)	C19—H19	0.9300
C7—N1	1.476 (3)	C20—H20A	0.9300
C7—H7A	0.9700	C20—H20B	0.9300
C7—H7B	0.9700	C21—C26	1.382 (3)
C8—N1	1.467 (3)	C21—C22	1.393 (3)
C8—C9	1.475 (4)	C21—S2	1.773 (2)
C8—H8A	0.9700	C22—C23	1.365 (4)
C8—H8B	0.9700	C22—Cl3	1.727 (3)
C9—C10	1.254 (4)	C23—C24	1.373 (4)
C9—H9	0.9300	C23—H23	0.9300
C10—H10A	0.9300	C24—C25	1.371 (4)
C10—H10B	0.9300	C24—H24	0.9300
C11—C16	1.386 (3)	C25—C26	1.384 (3)
C11—C12	1.391 (3)	C25—Cl4	1.728 (3)
C11—S1	1.784 (2)	C26—H26	0.9300
C12—C13	1.375 (3)	S1—O2	1.4226 (16)
C12—Cl1	1.724 (2)	S1—O1	1.4227 (16)
C13—C14	1.372 (4)	S1—N1	1.6011 (19)
C13—H13	0.9300	S2—O3	1.4249 (16)
C14—C15	1.378 (4)	S2—O4	1.4281 (16)
C14—H14	0.9300	S2—N2	1.6112 (18)
			
C6—C1—C2	120.9 (2)	N2—C17—C6	110.33 (18)
C6—C1—H1	119.6	N2—C17—H17A	109.6
C2—C1—H1	119.6	C6—C17—H17A	109.6
C3—C2—C1	119.0 (2)	N2—C17—H17B	109.6
C3—C2—C7	121.2 (2)	C6—C17—H17B	109.6
C1—C2—C7	119.7 (2)	H17A—C17—H17B	108.1
C2—C3—C4	120.2 (2)	N2—C18—C19	113.2 (2)
C2—C3—H3	119.9	N2—C18—H18A	108.9
C4—C3—H3	119.9	C19—C18—H18A	108.9
C5—C4—C3	120.4 (3)	N2—C18—H18B	108.9
C5—C4—H4	119.8	C19—C18—H18B	108.9
C3—C4—H4	119.8	H18A—C18—H18B	107.7
C4—C5—C6	120.7 (2)	C20—C19—C18	125.2 (3)
C4—C5—H5	119.7	C20—C19—H19	117.4
C6—C5—H5	119.7	C18—C19—H19	117.4
C5—C6—C1	118.8 (2)	C19—C20—H20A	120.0
C5—C6—C17	121.4 (2)	C19—C20—H20B	120.0
C1—C6—C17	119.7 (2)	H20A—C20—H20B	120.0
N1—C7—C2	109.52 (16)	C26—C21—C22	119.4 (2)
N1—C7—H7A	109.8	C26—C21—S2	117.30 (16)
C2—C7—H7A	109.8	C22—C21—S2	123.27 (18)
N1—C7—H7B	109.8	C23—C22—C21	120.2 (2)
C2—C7—H7B	109.8	C23—C22—Cl3	118.4 (2)
H7A—C7—H7B	108.2	C21—C22—Cl3	121.38 (19)
N1—C8—C9	111.8 (2)	C22—C23—C24	120.7 (3)
N1—C8—H8A	109.2	C22—C23—H23	119.6
C9—C8—H8A	109.2	C24—C23—H23	119.6
N1—C8—H8B	109.2	C25—C24—C23	119.4 (3)
C9—C8—H8B	109.2	C25—C24—H24	120.3
H8A—C8—H8B	107.9	C23—C24—H24	120.3
C10—C9—C8	125.5 (3)	C24—C25—C26	121.1 (2)
C10—C9—H9	117.3	C24—C25—Cl4	120.3 (2)
C8—C9—H9	117.3	C26—C25—Cl4	118.6 (2)
C9—C10—H10A	120.0	C21—C26—C25	119.2 (2)
C9—C10—H10B	120.0	C21—C26—H26	120.4
H10A—C10—H10B	120.0	C25—C26—H26	120.4
C16—C11—C12	119.06 (19)	O2—S1—O1	118.98 (11)
C16—C11—S1	116.79 (15)	O2—S1—N1	107.96 (10)
C12—C11—S1	124.15 (17)	O1—S1—N1	108.42 (10)
C13—C12—C11	119.9 (2)	O2—S1—C11	109.61 (10)
C13—C12—Cl1	117.98 (18)	O1—S1—C11	104.72 (10)
C11—C12—Cl1	122.09 (17)	N1—S1—C11	106.50 (9)
C14—C13—C12	121.1 (2)	O3—S2—O4	118.28 (10)
C14—C13—H13	119.5	O3—S2—N2	108.01 (9)
C12—C13—H13	119.5	O4—S2—N2	110.76 (10)
C13—C14—C15	119.0 (2)	O3—S2—C21	108.82 (10)
C13—C14—H14	120.5	O4—S2—C21	105.88 (10)
C15—C14—H14	120.5	N2—S2—C21	104.16 (10)
C16—C15—C14	121.0 (2)	C8—N1—C7	117.11 (18)
C16—C15—Cl2	118.46 (19)	C8—N1—S1	117.93 (15)
C14—C15—Cl2	120.56 (19)	C7—N1—S1	123.99 (14)
C15—C16—C11	119.9 (2)	C18—N2—C17	117.55 (18)
C15—C16—H16	120.0	C18—N2—S2	119.85 (15)
C11—C16—H16	120.0	C17—N2—S2	117.37 (14)
			
C6—C1—C2—C3	−1.0 (3)	C23—C24—C25—Cl4	−177.5 (2)
C6—C1—C2—C7	174.88 (18)	C22—C21—C26—C25	−0.1 (3)
C1—C2—C3—C4	1.1 (3)	S2—C21—C26—C25	178.15 (17)
C7—C2—C3—C4	−174.8 (2)	C24—C25—C26—C21	−1.3 (4)
C2—C3—C4—C5	−0.1 (4)	Cl4—C25—C26—C21	177.70 (16)
C3—C4—C5—C6	−0.9 (4)	C16—C11—S1—O2	−134.09 (17)
C4—C5—C6—C1	1.0 (4)	C12—C11—S1—O2	46.3 (2)
C4—C5—C6—C17	178.5 (2)	C16—C11—S1—O1	−5.38 (19)
C2—C1—C6—C5	0.0 (3)	C12—C11—S1—O1	175.01 (19)
C2—C1—C6—C17	−177.50 (18)	C16—C11—S1—N1	109.37 (17)
C3—C2—C7—N1	114.2 (2)	C12—C11—S1—N1	−70.3 (2)
C1—C2—C7—N1	−61.7 (2)	C26—C21—S2—O3	−125.84 (16)
N1—C8—C9—C10	123.0 (4)	C22—C21—S2—O3	52.3 (2)
C16—C11—C12—C13	3.0 (3)	C26—C21—S2—O4	2.29 (18)
S1—C11—C12—C13	−177.43 (19)	C22—C21—S2—O4	−179.54 (18)
C16—C11—C12—Cl1	−175.59 (17)	C26—C21—S2—N2	119.15 (16)
S1—C11—C12—Cl1	4.0 (3)	C22—C21—S2—N2	−62.7 (2)
C11—C12—C13—C14	−1.5 (4)	C9—C8—N1—C7	−65.1 (3)
Cl1—C12—C13—C14	177.1 (2)	C9—C8—N1—S1	104.1 (2)
C12—C13—C14—C15	−0.6 (4)	C2—C7—N1—C8	−61.9 (2)
C13—C14—C15—C16	1.2 (4)	C2—C7—N1—S1	129.61 (17)
C13—C14—C15—Cl2	−179.3 (2)	O2—S1—N1—C8	178.66 (16)
C14—C15—C16—C11	0.2 (4)	O1—S1—N1—C8	48.51 (19)
Cl2—C15—C16—C11	−179.29 (17)	C11—S1—N1—C8	−63.70 (18)
C12—C11—C16—C15	−2.3 (3)	O2—S1—N1—C7	−12.98 (19)
S1—C11—C16—C15	178.04 (18)	O1—S1—N1—C7	−143.13 (16)
C5—C6—C17—N2	−63.4 (3)	C11—S1—N1—C7	104.66 (17)
C1—C6—C17—N2	114.1 (2)	C19—C18—N2—C17	−73.6 (3)
N2—C18—C19—C20	116.5 (3)	C19—C18—N2—S2	80.2 (2)
C26—C21—C22—C23	1.2 (3)	C6—C17—N2—C18	−58.5 (3)
S2—C21—C22—C23	−176.92 (19)	C6—C17—N2—S2	147.10 (17)
C26—C21—C22—Cl3	−178.38 (16)	O3—S2—N2—C18	14.2 (2)
S2—C21—C22—Cl3	3.5 (3)	O4—S2—N2—C18	−116.77 (18)
C21—C22—C23—C24	−1.0 (4)	C21—S2—N2—C18	129.81 (18)
Cl3—C22—C23—C24	178.6 (2)	O3—S2—N2—C17	168.06 (17)
C22—C23—C24—C25	−0.3 (4)	O4—S2—N2—C17	37.1 (2)
C23—C24—C25—C26	1.5 (4)	C21—S2—N2—C17	−76.35 (19)

### Hydrogen-bond geometry (Å, °)

**Table:**

*D*—H···*A*	*D*—H	H···*A*	*D*···*A*	*D*—H···*A*
C8—H8A···O3^i^	0.97	2.59	3.422 (3)	144

Symmetry codes: (i) −*x*+1, −*y*, −*z*.
